# Protective effect of epigallocatechin-3-gallate (EGCG) on toxic metalloproteinases-mediated skin damage induced by Scyphozoan jellyfish envenomation

**DOI:** 10.1038/s41598-020-75269-1

**Published:** 2020-10-29

**Authors:** Du Hyeon Hwang, Hyunkyoung Lee, Indu Choudhary, Changkeun Kang, Jinho Chae, Euikyung Kim

**Affiliations:** 1 College of Veterinary Medicine, Gyeongsang National University, Jinju, 52828 Korea; 2 Institute of Animal Medicine, Gyeongsang National University, Jinju, 52828 Korea; 3 Marine Environmental Research and Information Laboratory, B1101, 17 Gosan-ro 148beon-gil, Gunpo-si, Gyeonggi-do, 15850 Korea

**Keywords:** Drug discovery, Health care, Medical research, Molecular medicine

## Abstract

Jellyfish stingings are currently raising serious public health concerns around the world. Hence, the search for an effective first aid reagent for the envenomation has been the goal of many investigators in the field. There have been a few previous reports of in vivo as well as in vivo studies suggesting the metalloproteinase activity of scyphozoan jellyfish venom, such as *N. nomurai* venom (NnV), plays a major role in the pathogenesis. These results have inspired us to develop a metalloproteinase inhibitor as a candidate for the treatment of Scyphozoan jellyfish envenomation. It has been previously demonstrated that the major polyphenol component in green tea, epigallocatechin-3-gallate (EGCG), can inhibit metalloproteinase activity of snake venoms. In fact, plant polyphenols as potential therapeutics have been shown to exert positive effects on neutralizing snake venoms and toxins. In the present study, we found that EGCG significantly inhibits the toxic proteases of NnV in a concentration-dependent manner. Human keratinocyte (HaCaT) and Human dermal fibroblast (HDF) cell culture studies showed that EGCG treatment can protect the cells from NnV-induced cytotoxicity which has been accompanied by the down-regulation of human matrix metalloproteinase (MMP)-2 and -9. Simulated rat NnV envenomation study disclosed that topical treatments with EGCG considerably ameliorated the progression of the dermonecrotic lesions caused by NnV. EGCG also reduced the activitions of tissue MMP-2 and MMP-9, which seem to be crucial players in the dermal toxic responses induced by NnV. Therefore, we propose that EGCG might be an effective therapeutic agent for the treatment of cutaneoous jellyfish symptoms.

## Introduction

Jellyfish envenomation has been a public health problem for a long time in all over the world, especially for Australia and tropical part in Southeast Asia^1–5^. However, the first aid or treatment for jellyfish envenomation has been far from being satisfactory. Jellyfish species have numerous numbers of cnidocytes in their tentacles, which are explosive cells containing an organelle called nematocyst. Each of these nematocysts encompasses a coiled, hollow, usually barbed thread with venom. Upon proper physical or chemical stimulation, such as human skin contact, nematocyst is rapidly discharged and everted thread penetrates into the skin and injects venom^6^. Jellyfish envenomation can be characterized by local effects (edema, burning sensation, erythematous eruption with small vesicle and necrosis) and sometimes the development of severe systemic reactions (cardiovascular alterations, hypovolemic shock, and respiratory collapse)^7–10^. Among these, cutaneous local tissue damage is most frequently observed in jellyfish stinging. Proper management against jellyfish stinging is very important in reducing the local tissue damages and complications after jellyfish envenomation. Interestingly, each of the first aid methods for jellyfish stinging shows an inconsistent efficacy. Considering the variety of venomous jellyfish species with different characteristics, these variations are not so surprising and the developments of customized treatments seem to be highly recommended rather than panacea treatment depending on the jellyfish types. Taking into account all these factors, however, the evidence based on scientific data is insufficient and the effectiveness of most of suggested treatments is not clear due to unavailability of randomized controlled trials^11^. The use of vinegar (or 4% acetic acid) is now commonly accepted as a first aid in the world for jellyfish stinging. However, it can stimulate nematocyst discharge in some jellyfish species, aggravating the complications, especially for Scyphozoa. According to our previous study, the treatment of 4% acetic acid against Scyphozoan *N. nomurai* jellyfish tentacles surprisingly caused massive nematocyst discharges, whereas Cubozoan *Carybdea brevipedalia* did not^12^. There are also some reports in which the application of vinegar increased nematocyst discharges in *Cyanea capillata*, *Lytocarpus philippinus* and *Chironex fleckeri*^13^. There are some commercial products that have been developed and proposed to be effective in preventing or relieving jellyfish stings^14,15^. Currently, there has been no previous report on intervening the envenomation caused by Scyphozoan jellyfish stingings. In the present study, we have investigated a novel therapeutic reagent which can neutralize and prevent the local tissue injuries caused by scyphozoan *N. nomurai* jellyfish venom.

Our previous studies have shown that scyphozoan jellyfish (if not all) venoms are abundant in metalloproteinase components, which dominantly contribute to the overall toxicity of the jellyfish^16^. It was also confirmed that toxic metalloproteases among the venom components play a major role in the pathogenesis of the affected skin in an animal model study^17^. Furthermore, venom metalloproteinase is well known to be “spreading factor” that help to easily diffuse target specific toxins into circulation by degrading the extracellular matrix and the connective tissues surrounding blood vessels^18,19^. Therefore, the inhibition of toxic metalloproteinases in jellyfish venom may not only protect the local tissue damages, but also increase the survival rate of the victim by suppressing the systemic effect of the venoms.

Recently, the application of traditional medicines for treating envenomation has been attracting the attention of many investigators due to the benefits of easy availability, safety and neutralizing efficacy against broad ranges of natural toxins. Polyphenols, which are distributed widely in the plant kingdom, can interact with the enzymes from snake venoms and act as an antidote^20–22^. Fraction of Persimmon tannin exerted a very strong inhibitory effect on the catalytic activity of Chinese cobra venom phospholipase A_2_ (PLA_2_), and alleviated the myotoxicity, neurotoxicity and lethality induced by the venom PLA_2_ in vivo^23,24^. Polyphenol from the aqueous extracts of *Pentace burmanica*, *Pithecellobium dulce*, *Areca catechu* and *Quercus infectoria* showed anti-venom activities through either selectively blocking the nicotinic acetylcholine receptor or non-selectively precipitating the venom proteins^25^. Epigallocatechin-3-gallate (EGCG) belongs to the major component of green tea polyphenols. It has been reported to inhibit several clinically important enzymes and cure pathological disorders^25–29^. Many studies have suggested that EGCG can mitigate the metalloproteinase expression in target cells, which is induced by several agents, such as carcinogens, ultraviolet A, phorbol myristate acetate, interleukin and tumor necrosis factor-alpha^30–33^. Furthermore, EGCG or its structural analogues has shown the inhibitory effects on cutaneous inflammation and infection-induced inflammatory reactions^34–36^. Several studies have even revealed the neutralization of the toxic effects induced by snake venoms using either polyphenol compounds or polyphenol-containing plant extracts^25,37,38^. In the present work, we have investigated the potential therapeutic/preventive effects of EGCG on NnV-induced dermal toxicity using in vitro and in vivo models.

## Results

### Effect of EGCG on NnV-induced proteolytic activity

**Figure 1:**
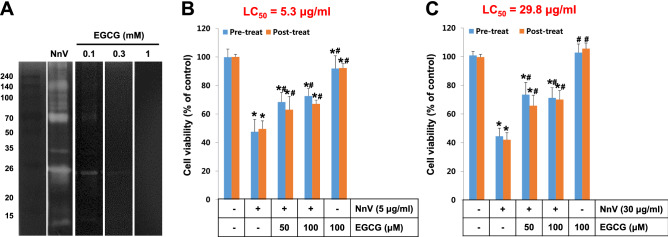
Inhibitory effects of EGCG on the proteolytic activity and the cytotoxicity of NnV. (**A**) NnV (4 μg) was loaded and run on SDS-PAGE, followed by gelatin zymography in reaction buffers containing various concentrations of EGCG (0.1, 0.3 and 1 mM). To examine the effect of EGCG on NnV-induced cell death, HaCaT cells (**B**) or HDF cells (**C**) were treated with the indicated concentrations of EGCG before or after NnV exposure (30 μg/ml) with 30 min. interval. Data represent as mean ± SD from three independent experiments performed on different well replicates. *p < 0.01 compared with vehicle control. ^#^p < 0.01 compared with NnV alone.

According to our previous study, there are abundant metalloproteinase activities in NnV, which are largely contributing to the venom toxicity^16^. Hence, the inhibition of metalloproteinase of NnV has been proposed as a therapeutic target against the poisoning symptoms induced by *N. nomurai* stinging^17^. To verify if EGCG can modulate the metalloproteinases of NnV, gelatin zymography has been performed (Fig. 1A). As shown by the white bands in zymography, NnV alone showed prominent gelatinolytic activity, which was almost completely suppressed in the presence of EGCG in a concentration-dependent manner. Since NnV stinging may cause dermal tissue injury, it has also been evaluated if EGCG reduce NnV-associated dermal toxicity using human skin cell lines (Fig. 1B,C). The results elucidated that NnV exposure suppressed the cell viabilities of HaCaT (keratinocyte) and HDF (fibroblast) cells with LC_50_ values of 5.3 μg/ml and 29.8 μg/ml, respectively. It implicates that NnV has a higher potency on HaCaT cells than HDF cells. In a pre-treatment study, EGCG was added for 1 h before the treatment of NnV. The NnV-induced cytotoxicity was reduced approximately 1.5 folds by pre-treatment of EGCG in both cells. Interestingly, the post-treatment of EGCG for 1 h after the treatment of NnV has also some protective effects, although the viabilities in HaCaT and HDF cells were slightly less than those of pretreatment. These results suggest that the application of EGCG-containing product on the sting area of patient skin might be effective as a first aid reagent against jellyfish stings.

### Inhibitory effects of EGCG on NnV-induced activation of skin cell endogenous MMPs

**Figure 2:**
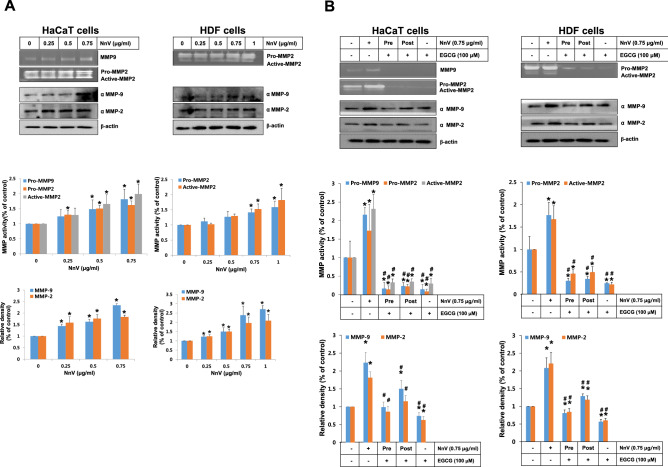
Modulatory effect of EGCG on NnV-induced activation of endogenous MMPs. (**A**) Indicated concentrations of NnV were exposed to HaCaT or HDF cells for 24 h. (**B**) EGCG was also treated for the both cells, either before (Pre) or after (Post) the NnV exposure (0.75 μg/ml) with 1 h interval. After collecting the mediums and the cell lysis samples, they were loaded on SDS-PAGE and then evaluated with gelatin zymography and Western blotting . Data represent as mean ± SD from three independent experiments. *p < 0.01 compared with vehicle control. ^#^p < 0.01 compared with NnV alone.

Upon the exposure to the low (not mortal) levels of NnV, MMP-2 and -9 secretions from HaCaT cells were concentration-dependently increased by NnV, whereas MMP-9 in HDF cells was not observed by gelatin zymography method probably due to its low level of activity (Fig. 2A). Based on Western blot results, the protein expressions of MMP-2 and -9 were increased over 2 folds by the treatment of NnV (0.75 μg/ml) comparing with the control group cells. In HDF cells, the expression of MMP-2 was augmented in a concentration-dependent manner, but MMP-9 were more intensely detected in NnV 0.75 than 1 μg/ml. Therefore, the optimal concentration of NnV (0.75 μg/ml) in terms of MMPs activation was chosen for subsequent experiments in these cells. Next, we investigated the effects of EGCG on the activations and expressions of MMPs in the cells during NnV exposure. Both pre-treatment and post-treatment of EGCG dramatically suppressed the activities of MMP-2 and -9, which were induced by the exposure of HaCaT and HDF cells to NnV (Fig. 2B).

### Protective effect of EGCG on NnV-induced apoptotic deaths of human skin cells

**Figure 3:**
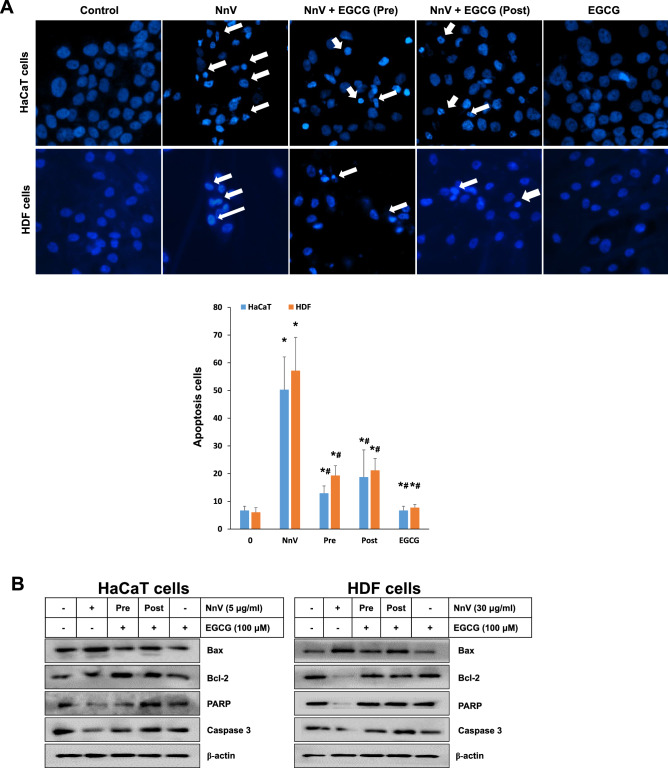
Protective effects of EGCG on NnV-induced apoptotic deaths of human skin cells. Both HaCaT cells and HDF cells were treated with EGCG before (Pre) or after (Post) the NnV exposure (30 μg/ml). (**A**) Apoptotic cell morphological alterations were visualized by DAPI staining and the representative images were captured with a microscope (Thermo) using a 200 × objective. (**B**) The expression levels of apoptosis-related proteins were analyzed by Western blotting using their specific monoclonal antibodies. Data represent as mean ± SD from three independent experiments. *p < 0.01 compared with vehicle control. ^#^p < 0.01 compared with NnV alone.

Since NnV exposure brought about cell death in both HaCaT and HDF cells, they were examined whether the cells undergo apoptosis by the exposure to NnV in the absence or the presence of of EGCG. NnV exposures to HaCaT and HDF cells unveiled nucluear shrinkage and fragmentation in both cells via DAPI staining (Fig. 3A). Simultaneously, the expressions of Bcl-2, procaspase-3 and PARP were dramatically decreased, whereas the level of Bax was increased following NnV exposure in both cell types (Fig. 3B). However, EGCG treatments distinctly reduced the apoptotic cell deaths as well as the expressions of apoptosis-related proteins. The protection by EGCG was very effective, regardless of its treatment methods (Pre- or Post-) in both cell types.

### Protective effect of topical EGCG application on the affected skin area of NnV-injection

**Figure 4:**
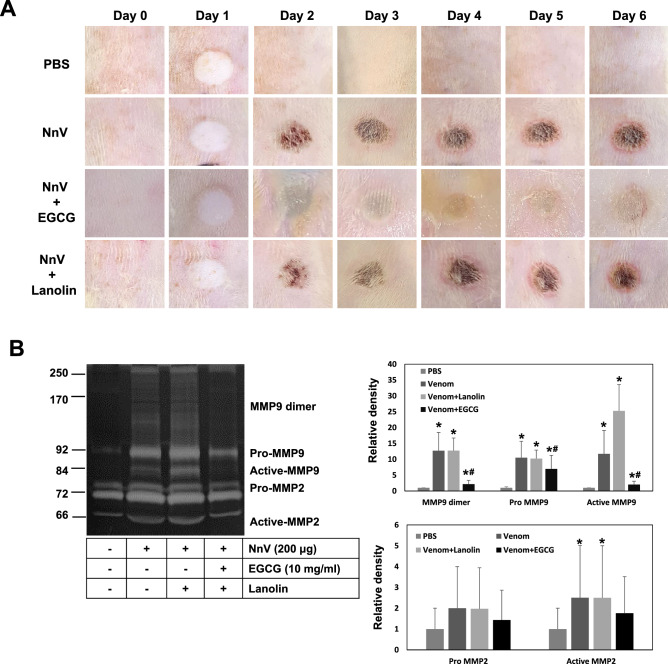
Protective effect of topical EGCG application on NnV-induced dermonecrotic injury. (**A**) The representative dermonecrotic changes were photographed daily basis for the entire period of experiment. (**B**) Upon the completion of the treatments, the tissue MMPs activities were examined from the skins of injection site using gelatin zymography, and then quantified for the secreatory levels of MMP-2 and -9. Data represent as mean ± SD from the three fields. *p < 0.01, compared with PBS group and ^#^p < 0.01, compared with NnV group.

SD rats were intradermally inoculated with either phosphate buffered saline (PBS) or NnV (200 μg) and then testing substances (EGCG in lanolin cream or lanolin cream alone) were topically applied on the injection sites once daily for 6 days (Fig. 4A). Unlike PBS group (vehicle), NnV group (positive control) clearly showed degenerative changes on injection site from day 2 and the skin site has continuously aggravated to dermonecrotic lesion during the experiment periods. On the other hand, EGCG-treated group exhibited a noticeable reduction of dermonecrosis from its early stage (Day 2) of venom reaction. The results demostrated EGCG has an excellent protective effect comparing with either lanolin cream alone or no topical treatment. Upon the completion of the treatments (Day 6), gelatin zymography was performed using the skin tissues of injection site to evaluate the association between NnV-induced dermal lesions and their endogenous tissue MMP levels. NnV alone significantly increased the activities of pro-, and active-MMP-9 as well as pro- and active-MMP-2 compared with vehicle (PBS) group (Fig. 4B). Especially, pro- and active-MMP-9 in tissue were extremely provoked by NnV. The topical application of lanolin cream alone did not affect the dermonecrotic lesion as well as MMP-2 and -9 activities. On the other hand, EGCG in lanolin cream dramatically suppressed the NnV-induced dermonecrotic symptoms as well as the tissue MMP-2 and -9 activities, suggesting the potential protective effect of topical EGCG application on the skin site of jellyfish stinging.

### Histopathological assessment of skin damages

**Figure 5:**
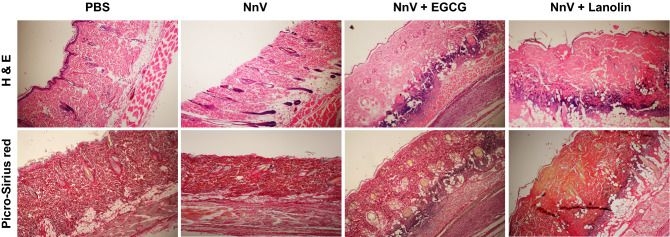
Protective effects of topical EGCG application against NnV-induced dermonecrosis. On day 6, the skin lesions were collected and then fixed in 10% neutralized formalin solution for observing the histopathological alterations upon the treatment of EGCG on NnV-induced dermal necrosis. The tissues were stained with Hematoxylin and Eosin (H & E) as well as Picro-sirius red staining.

NnV-injection induced the characteristic tissue damages, including loss of epidermis, destruction of collagenous fiber, edema formation, neutrophil infiltration and hemorrhage comparing with PBS control group. On the other hand, EGCG-treatment markedly reduced hemorrhage and neutrophil infiltration produced by NnV exposure. EGCG also prevented the loss of epidermis and collagenous fibers. These results suggest that dermonecrotic lesions by NnV can be almost completely blocked by EGCG treatment. Furthermore, as evidenced by Picro-sirius red assay, PBS group showed dense and regulatory collagen fibers in dermis, whereas NnV-exposure resulted in not only the destruction of collagen fibers in dermis but also only a few weak signals of fibrillar fibers, indicating poor regeneration after envenomation-induced tissue damages (Fig. 5). The topical application of lanolin alone exhibited a good amount of fibrillar fibers, suggesting that lanolin alone is not enough but may moderately help or at least partially prevent the dermal injury induced by NnV. In conclusion, the tropical application of EGCG was most effective for preventing the destruction of epithermal layers and regenerating collagenous fiber for the skin sites of jellyfish stinging.

## Discussion

There has been an increase of jellyfish stinging accidents in various coastal areas of the world^14^. Although not all species of jellyfish are poisonous, the hazard by jellyfish should not be ignored because stings of some species induce serious toxic symptoms^1,3^. Jellyfish sting commonly induced cutaneous damage, which is reported to be caused by metalloproteinase component. Venom metalloproteinase is reported to play an important roles in the local pathogenesis, such as local myonecrosis, dermonecoris, and inflammatory reaction^39,40^. According to the analyses of transcriptome and proteome in schyphozoan jellyfish venom, metalloproteinase is one of the predominant components and largely contributes to the development of pathgenesis induced by the venom^41–45^. Snake venom metalloproteinases degrade extracellular matrix (ECM) that cause local hemorrhage and tissue damage^46^.

Polyphenols are known to have depressing effects on proteolytic activity due to their ability to bind and precipitate proteins. The toxic effects of numerous toxins, such as snake venom, botulinum toxin, tetanus toxin, and Shiga toxin have been subsided by polyphenol^23,36,47–49^. Among the polyphenols, condensed tannins (catechin, epicatechin and EGCG) are excellent precipitator, which directly form the complexes with proteins for the binding of tannins, including phenolic residues and polar groups of protease^25^. Condensed tanins also can be interacted with zinc and calcium in the active sites of metalloproteinase in venom, leading to indirectly inhibiting the enzymatic activity^50^. Thus, EGCG is of potential value for treating patients stung by jellyfish which have metalloproteinase-like enzymes in their venoms. In addition to venom metalloproteinase activity, local tissue damage has been attributed to activations of endogenous MMPs^17^. Endogenous MMPs play the important roles for the degradation of connective tissues and influence of inflammatory process in both physiological and pathological conditions^51^. Activated MMPs induce the recruitment of neutrophils, the destruction of connective tissues and the participation of the first step in wound-healing process. Particularly, MMP-2 and -9 are responsible for mainly degrading type IV and V collagens, the major components in extracellular matrix. MMP-2 has a key role in the degradation of ECM and produced by fibroblasts under normal condition. Instead, MMP-9 is especially most provoked by stimulatory factors such as inflammatory cytokines, carcinogens and growth factors and is secreted in the initiation as well as the end stages of inflammatory responses, and the migration of leukocytes. Based on the present work, EGCG has inhibitory effects on NnV-induced MMP-2 and -9 activations, but not lanolin alone. Furthermore, EGCG prevented the development of dermal alterations, including the destructions of connetive tissue, and muscle fiber and the loss of epidermis through decresing the activities of MMP-2 and -9. Being consistent with the in vitro data, EGCG reduced or abrogated NnV-induced MMP-2 and -9 activities and expressions. These data suggest that the beneficial effects of EGCG involve the reduction of expressions/secretions of MMPs and consequently the decrease in local tissue damage. There were also predominant infiltration of neutrophil, increasing phagocytic capacity and recruitment of monocytes after the NnV inoculation. In contrast, the treatement of EGCG subsided the levels of neutrophil accumulations and hemorrhages, while promoted the regeneration of damaged sites. Therefore, the protective effects of EGCG on NnV-induced local tissue damages seem to be associated with the regulation of inflammatory mediators, such as cytokines, endogenous MMPs as well as the migration of leukocytes. Furthermore, NnV also provoked the cell deaths in HaCaT and HDF cells, whereas EGCG protected cells from NnV-associated cytotoxicity and dermonecrotic pathogenesis.

## Materials and methods

### Chemicals and reagents

Dulbecco’s modified Eagle’s Medium (DMEM), Fibroblast basal medium (FBM), Fetal bovine serum (FBS), Bovine serum albumin (BSA), penicillin, streptomycin and trypsin were purchased from Gibco-BRL (Grand Island, NY, USA). Fibroblast growth medium-2 (FGM-2) were purchased from Lonza (Walkersville, MD, USA). Dimethyl sulfoxide (DMSO) and 3-(4,5-dimethylthiazol-2-yl)-2,5-diphenyltetrazolium bromide (MTT) were from Sigma-Aldrich Inc. (St. Louis, MO, USA). Antibodies for MMP-2, and MMP-9, Bax, Bcl-2, PARP, caspase-3 and *β*-actin were obtained from Bioword technology Inc. (St Louis Park, MN, USA). All other reagents used were of the purest grade available.

### Jellyfish collection and preparation

The specimens of *N. nomurai* jellyfish were captured from the Korea Strait along the coasts of Geoje in September, 2012. Only tentacles were collected in ice. Nematocysts were isolated from the dissected tentacles as described by a Bloom method with slight modification^52^. In brief, dissected tentacles were rinsed with cold seawater to remove debris. The tentacles were placed in three volumes of cold seawater for 24 h with gentle swirling for 1 h once daily at 4 °C. After autolysis for 24 h at 4 °C, the supernatant was collected and centrifuged at 4000×*g* for 10 min and the settled material was resuspended in fresh seawater and set for autolysis for 24 h. This process was repeated for 3 days. Finally, the undischarged nematocysts were collected, lyophilized and stored at − 70 °C until use.

### Venom extraction and preparation

Venom was extracted from the freeze-dried nematocysts using the technique described Lee et al.^53^. In brief, venom was extracted from 50 mg of lyophilized nematocyst powder in 1 ml of cold phosphate buffered saline (PBS, pH 7.4, 4 °C) using glass beads. This mixture was shaken at 3000 rpm for 30 s, which was repeated for ten times with intermittent cooling on ice. The venom extracts were then transferred to a new Eppendorf tube and centrifuged (15,000 g) at 4 °C for 30 min. The supernatant was used as jellyfish venom extract (NnV) for the present study. Protein concentration of NnV was determined by Bradford method (Bio-Rad, CA, USA) and NnV was used based on its protein concentration^54^.

### Cell culture and Cell viability

HaCaT cells were cultured in DMEM supplemented with antibiotics and 10% FBS at 37 °C in a humidified atmosphere with 95% air and 5% CO_2_. HDF cells were cultured in FGM-2 using same conditions. The cell viability was measured by MTT reduction assay in the absence or the presence of indicated concentrations of NnV and/or EGCG as previously described^16^. In protective effect study, Cells were pre-treated with EGCG for 1 h before being tested cytotoxicity of NnV and were post-treated with EGCG after treating NnV in 1 h later.

### Gelatin zymography

Gelatin was usd as substrate for the proteolytic zymography assay as described by Lee et al.^16^. In brief, Cells were seeded 1 × 10^5^ cells/well in 6-well and aloowed to grow to confluence for 24 h and maintained in complete media. The cells were washed with PBS and incubated with NnV in serum-free media for 24 h. The supernatant was collected and mixed with non-reducing sample buffer, then electrophoresed in 10% SDS gel containing 0.1% gelatin. After the electrophoresis, the gel was washed for 30 min twice with 2.5% Triton X-100 and incubated for additional 18 h at 37 °C for the enzymatic reaction in zymography reaction buffer. The gel was then stained with Coomassie blue R-250 and destained. To investigate inhibitory effect of EGCG on proteolytic activity of NnV, NnV mixed with non-reducing sample buffer and then loaded 4 μg/ml in gelatin zymogel.

### Western blotting

Western blotting assay was performed as previously described^53^. Cells treated with NnV in the pretreatment and post-treatment of EGCG for 24 h were rinsed twice with ice-cold PBS. The treated cells were collected by scraping with 150 μL of RIPA buffer containing protease inhibitor cocktail. The sample proteins (20 μg) were run on 12% SDS–polyacrylamide gel, transferred to PVDF membranes and subsequently subjected to immunoblot analysis using specific primary antibodies for overnight at 4 °C. After washing, the membranes were incubated with horseradish peroxidase-conjugated secondary antibody (Cell Signaling Technology, Beverly, MA) for 1 h at room temperature. The blots were visualized by using the enhanced chemiluminescence method (ECL, Amersham Biosciences, UK) and analyzed using Chemi doc XRS (Bio-Rad, C.A. USA). Densitometry analysis was performed with a Hewlett-Packard scanner and NIH Image software (Image J).

### Dermal toxicity test

Sprague Dawley (SD) rats at 7 weeks of age were purchased from the Samtako Incorporation (Seoul, Korea). The animal protocol used in this study was approved by the Institutional Animal Care and Use Committee of the Gyeongsang National University at Jinju, and the animal protocol number is: GNU-151006-B0055. PBS or NnV 200 μg was intradermally injected into the shaved part on the dorssum of SD rats. After NnV injection, the rats were randomly grouped for experiment (5 rats/group) and the topical applications of EGCG (10 mg/mL) in lanolin cream or lanolin cream alone were initiated. The topical treatments were repeated once a day for 6 consecutive days. PBS group (negative control) was injected with PBS and NnV group (positive control) was injected with NnV with no additional treatment. The skin lesions were monitored daily basis and photographed prior to the following treatment.

### Tissues sampling and histological analysis

After 6 days, the animals were sacrificed by terminal exsanguination after being anesthetized with isofluran. Skin tissues of injection site to evaluate were homogenized by sonicator in 0.5 ml of Tris–HCL buffer. The supernatant obtained after the centrifugation of the homogenate (4 °C, 10,000 rpm, and 10 min) was removed and used for gelatin zymography assay. The obtained skin sections were fixed in 10% neutralized formalin solution. Then, the tissues were dehydrated and embedded in paraffin. The tissue blocks were sectioned, and stained with hematoxylin and eosin (H&E) for the observation of histological changes. To detect any regeneration from damaged tissues, Picro-sirius red staining was performed.

### Statistical analysis

The results are expressed as the mean ± standard deviation (S.D.). A paired Student’s *t*-test was used to assess the significance of differences between two mean values. P < 0.01 was considered to be statistically significant.

### Ethical approval and informed consent

All experimental protocols were approved by the Institutional Animal Care and Use Committee of the Gyeongsang National University. The methods were carried out in accordance with the relevant guidelines and regulations.

## Supplementary information

Supplementary Information 1.

Supplementary Information 2.

Supplementary Information 3.

Supplementary Information 4.

Supplementary Information 5.

Supplementary Information 6.

Supplementary Information 7.
