# Supplementary figures and images for: Protective effect of epigallocatechin-3-gallate (EGCG) on toxic metalloproteinases-mediated skin damage induced by Scyphozoan jellyfish envenomation
Source: Sci Rep. 2020 Oct 29;10:18644. doi: 10.1038/s41598-020-75269-1 (PMC7596074; doi:10.1038/s41598-020-75269-1)

## Slide 1
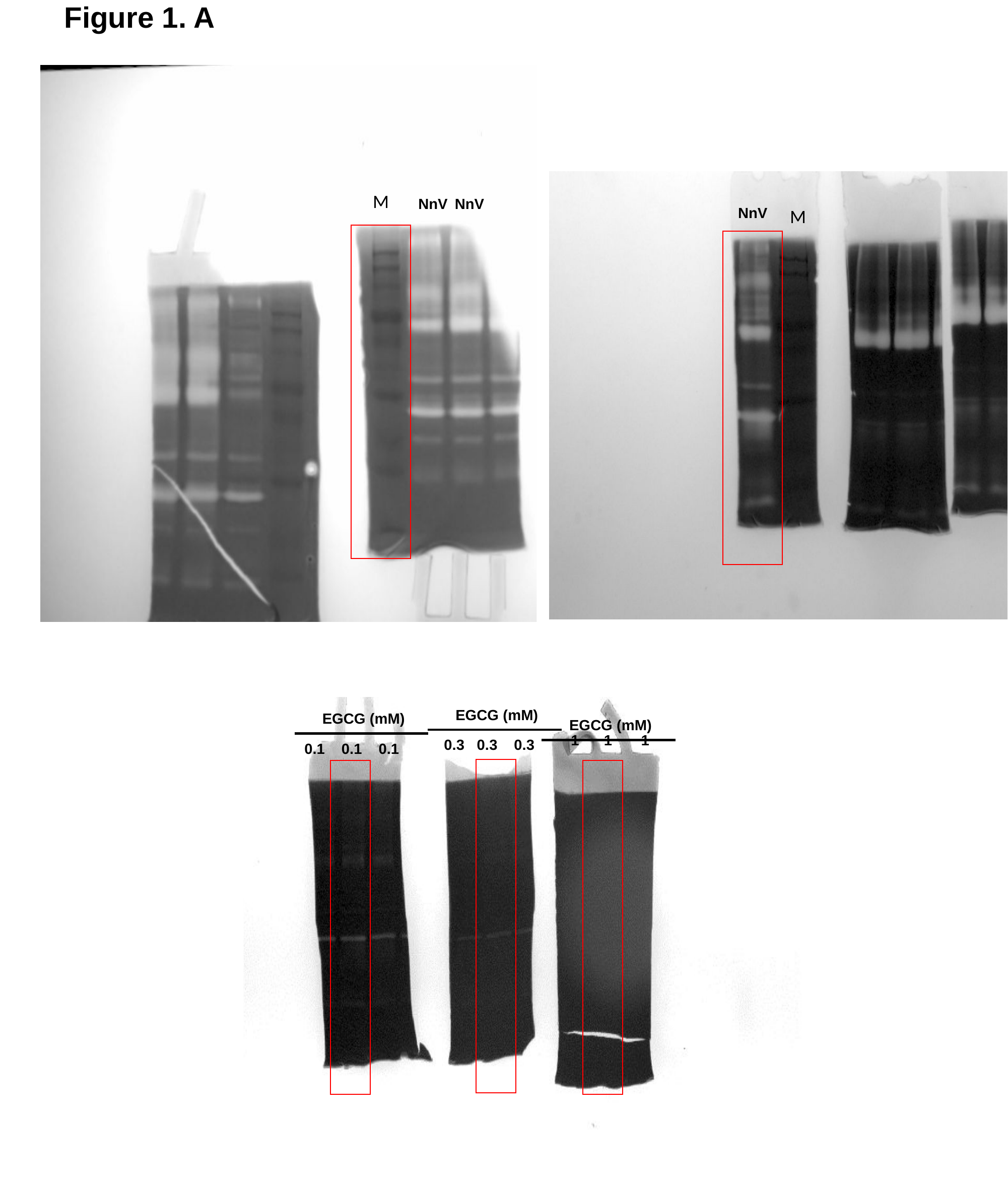

Figure 1. A
M
NnV
NnV
NnV
M
EGCG (mM)
EGCG (mM)
EGCG (mM)
1 1 1
0.3 0.3 0.3
0.1 0.1 0.1

Supplement: Supplementary file 1 — Supplementary Information 1. [file 41598_2020_75269_MOESM1_ESM.pptx]

## Slide 1
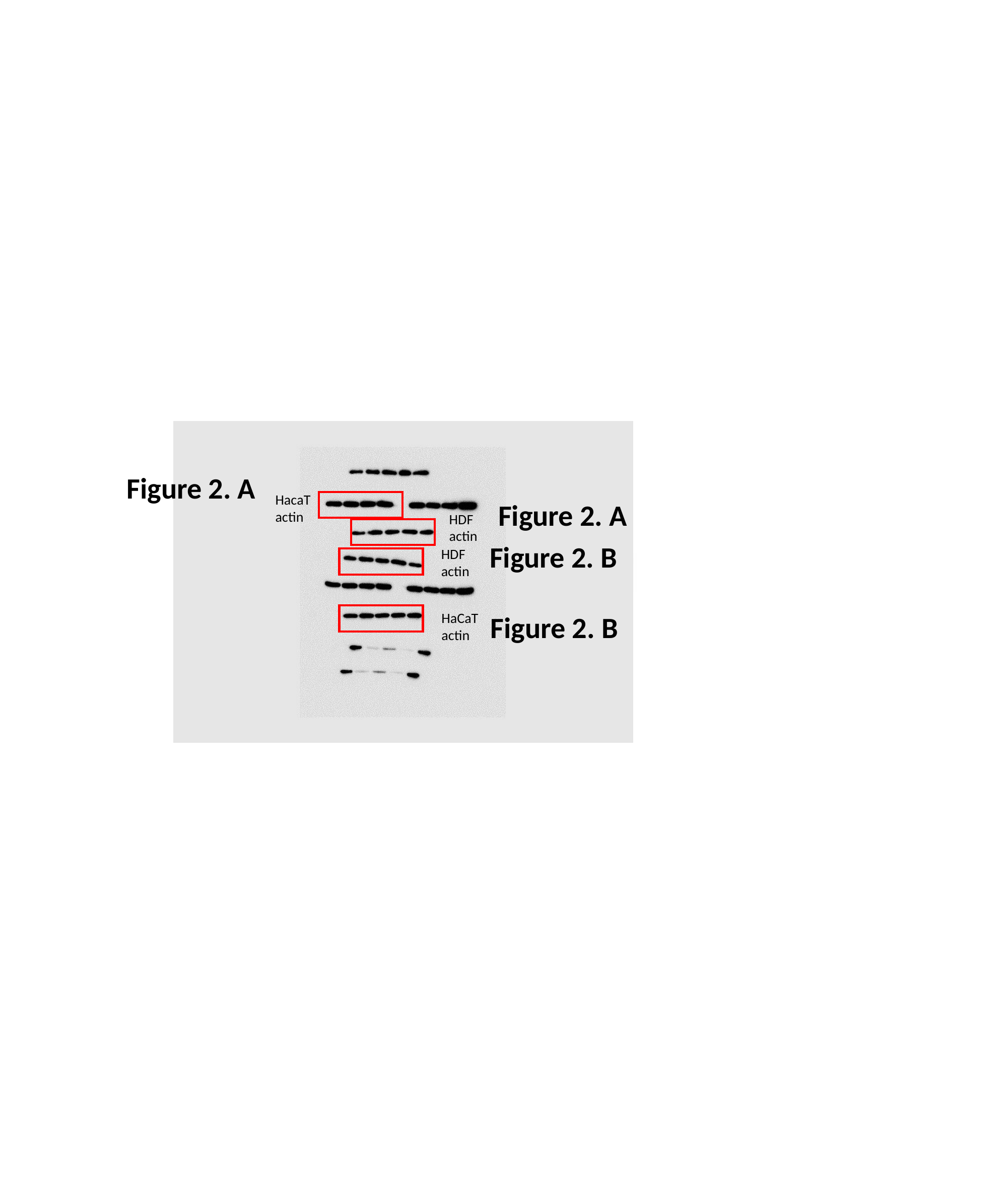

Figure 2. A
HacaT
actin
Figure 2. A
HDF
actin
Figure 2. B
HDF
actin
HaCaT
actin
Figure 2. B

Supplement: Supplementary file 3 — Supplementary Information 3. [file 41598_2020_75269_MOESM3_ESM.pptx]

## Slide 1
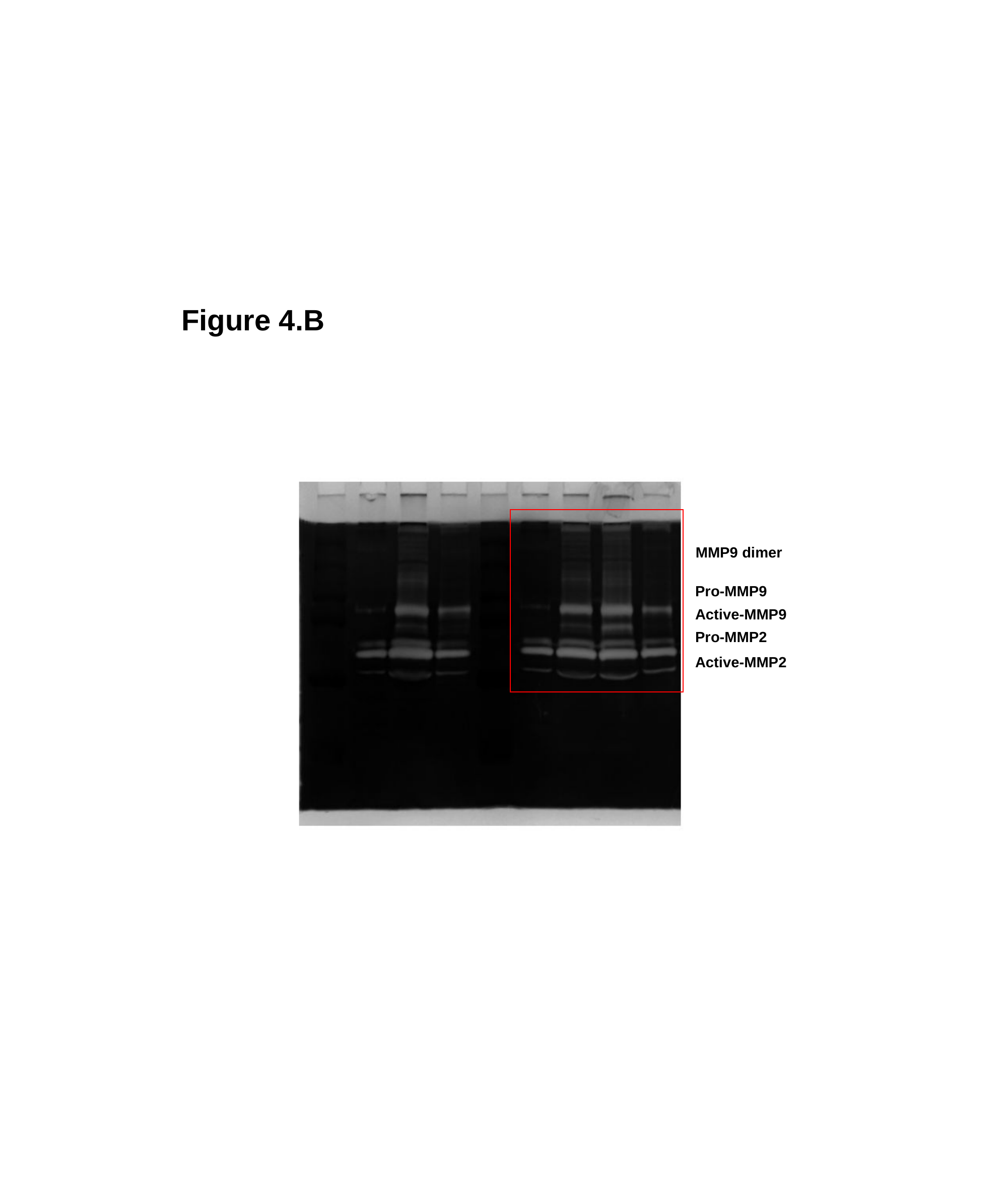

Figure 4.B
MMP9 dimer
Pro-MMP9
Active-MMP9
Pro-MMP2
Active-MMP2

Supplement: Supplementary file 6 — Supplementary Information 6. [file 41598_2020_75269_MOESM6_ESM.pptx]
