# Supplementary material for: Protective effect of epigallocatechin-3-gallate (EGCG) on toxic metalloproteinases-mediated skin damage induced by Scyphozoan jellyfish envenomation
Source: Sci Rep. 2020 Oct 29;10:18644. doi: 10.1038/s41598-020-75269-1 (PMC7596074; doi:10.1038/s41598-020-75269-1)
Supplement: Supplementary file 2 — Supplementary Information 2. [file 41598_2020_75269_MOESM2_ESM.pptx]

## Slide 1
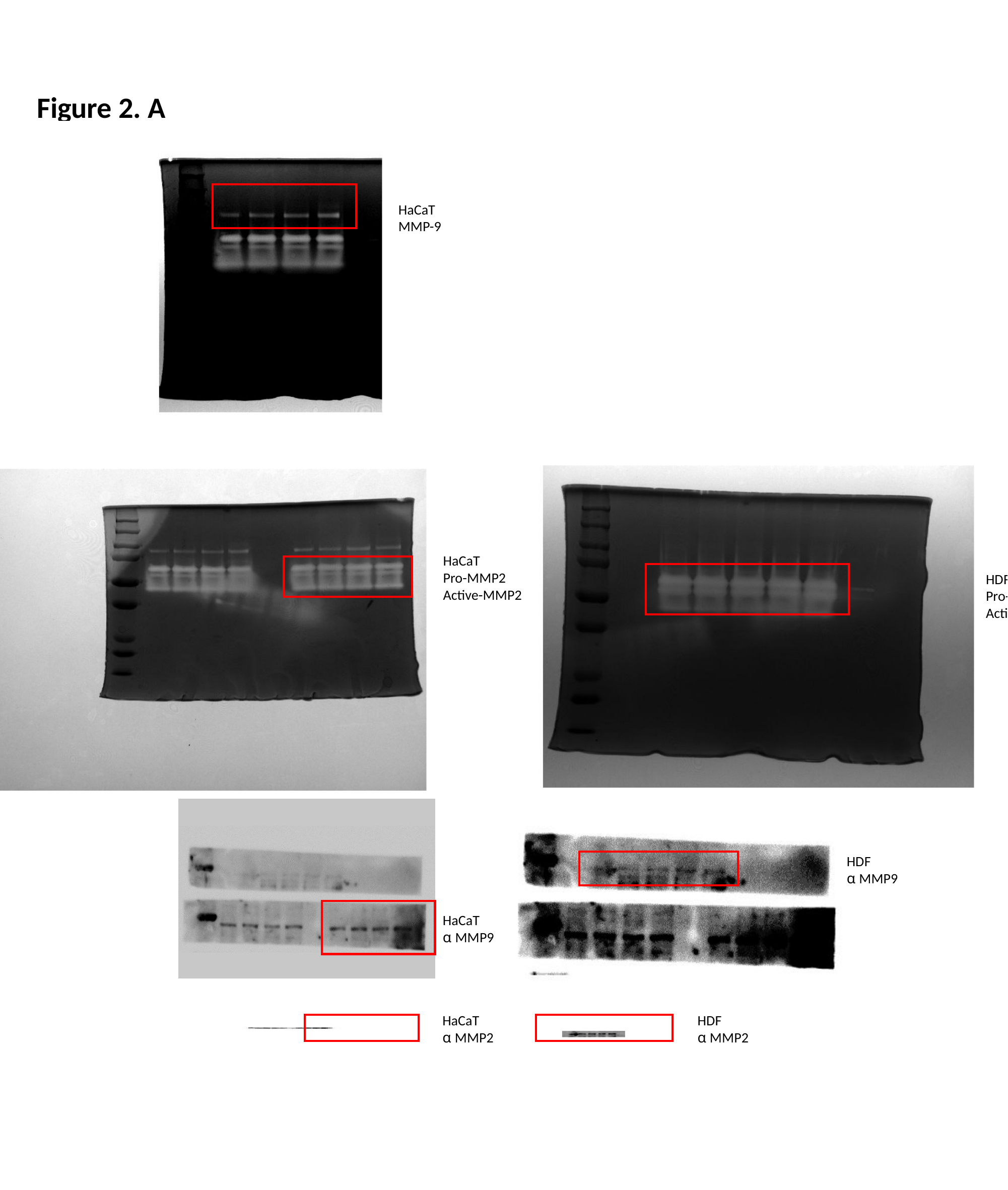

Figure 2. A
HaCaT
MMP-9
HaCaT
Pro-MMP2
Active-MMP2
HDF
Pro-MMP2
Active-MMP2
HDF
α MMP9
HaCaT
α MMP9
HDF
α MMP2
HaCaT
α MMP2
