# Supplementary material for: Protective effect of epigallocatechin-3-gallate (EGCG) on toxic metalloproteinases-mediated skin damage induced by Scyphozoan jellyfish envenomation
Source: Sci Rep. 2020 Oct 29;10:18644. doi: 10.1038/s41598-020-75269-1 (PMC7596074; doi:10.1038/s41598-020-75269-1)
Supplement: Supplementary file 4 — Supplementary Information 4. [file 41598_2020_75269_MOESM4_ESM.pptx]

## Slide 1
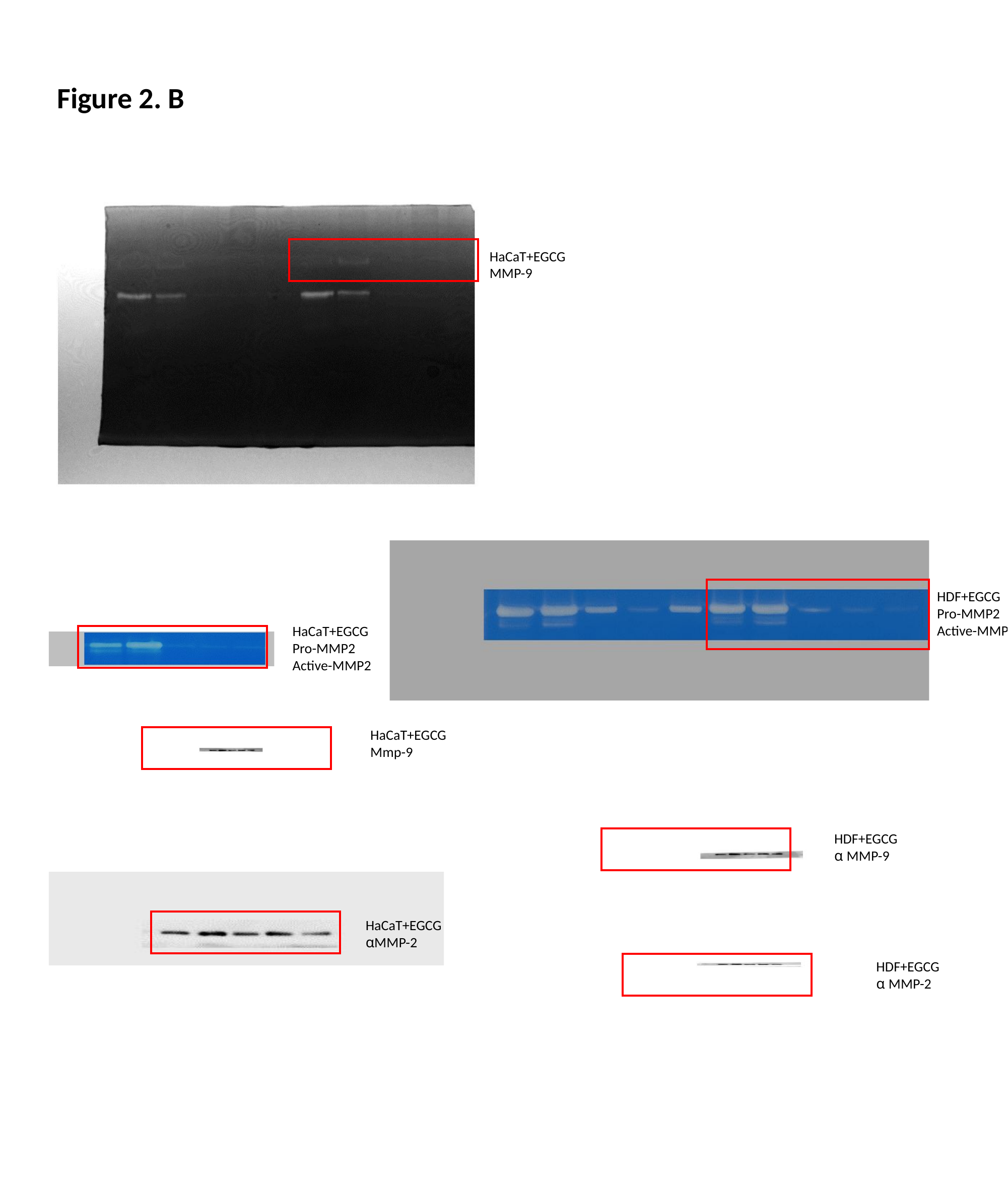

Figure 2. B
HaCaT+EGCG
MMP-9
HDF+EGCG
Pro-MMP2
Active-MMP2
HaCaT+EGCG
Pro-MMP2
Active-MMP2
HaCaT+EGCG
Mmp-9
HDF+EGCG
α MMP-9
HaCaT+EGCG
αMMP-2
HDF+EGCG
α MMP-2
