# Supplementary material for: Protective effect of epigallocatechin-3-gallate (EGCG) on toxic metalloproteinases-mediated skin damage induced by Scyphozoan jellyfish envenomation
Source: Sci Rep. 2020 Oct 29;10:18644. doi: 10.1038/s41598-020-75269-1 (PMC7596074; doi:10.1038/s41598-020-75269-1)
Supplement: Supplementary file 5 — Supplementary Information 5. [file 41598_2020_75269_MOESM5_ESM.pptx]

## Slide 1
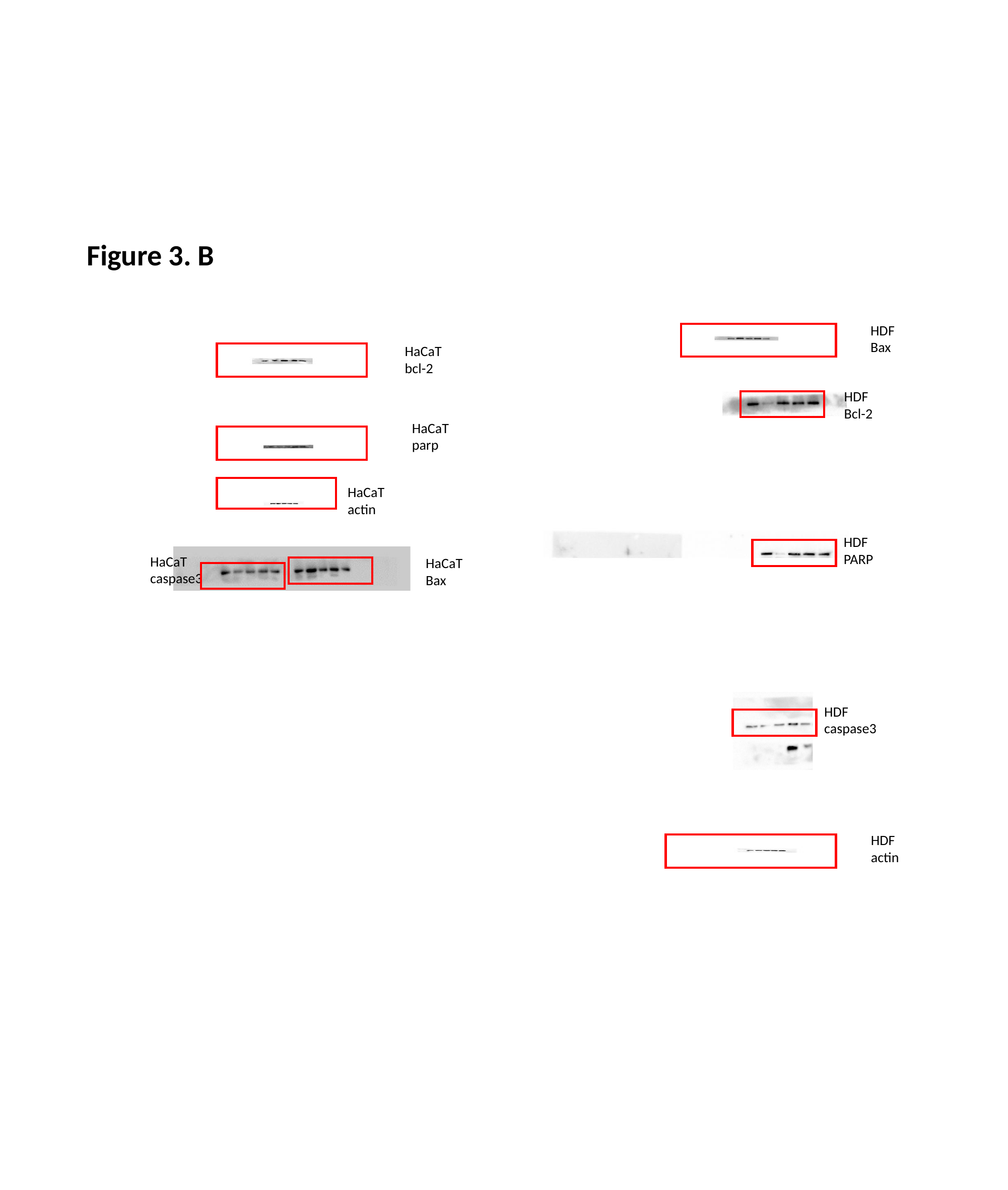

Figure 3. B
HDF
Bax
HaCaT
bcl-2
HDF
Bcl-2
HaCaT
parp
HaCaT
actin
HDF
PARP
HaCaT
caspase3
HaCaT
Bax
HDF
caspase3
HDF
actin
