# Supplementary material for: Protective effect of epigallocatechin-3-gallate (EGCG) on toxic metalloproteinases-mediated skin damage induced by Scyphozoan jellyfish envenomation
Source: Sci Rep. 2020 Oct 29;10:18644. doi: 10.1038/s41598-020-75269-1 (PMC7596074; doi:10.1038/s41598-020-75269-1)
Supplement: Supplementary file 7 — Supplementary Information 7. [file 41598_2020_75269_MOESM7_ESM.docx]

**Protective effect of epigallocatechin-3-gallate (EGCG) on toxic metalloproteinases-mediated skin damage induced by Scyphozoan jellyfish envenomation**

Du Hyeon Hwang^1,2^, Hyunkyoung Lee^1^, Indu Choudhary^1^ , Changkeun Kang^1,2^, Jinho Chae^3,§^ and Euikyung Kim^1,2,§^

^1^College of Veterinary Medicine, Gyeongsang National University, Jinju, 52828, Korea; E-Mails: [pooh9922@hanmail.net](mailto:pooh9922@hanmail.net) (D. H. H.) https://orcid.org/0000-0002-7228-3340, leehy@gnu.ac.kr (H. L.), [induchoudhary2u@gmail.com](mailto:induchoudhary2u@gmail.com) (I.C.), [ckkang@gnu.ac.kr](mailto:ckkang@gnu.ac.kr) (C. K.) https://orcid.org/0000-0002-5114-5096, [ekim@gnu.ac.kr](mailto:ekim@gnu.ac.kr) (E. K.) https://orcid.org/0000-0003-3356-3072

^2^Institute of Animal Medicine, Gyeongsang National University, Jinju, 52828, Korea

^3^Marine Environmental Research and Information Laboratory, B1101, 17 Gosan-ro 148beon-gil, Gunpo-si, Gyeonggi-do 15850, Korea; [jinhochae@gmail.com](mailto:jinhochae@gmail.com) (J. C.) <https://orcid.org/0000-0002-7229-0700>
